# Unveiling the Silent Struggle: A Teen’s Battle With Heart Failure Masking Takayasu Arteritis

**DOI:** 10.7759/cureus.50154

**Published:** 2023-12-08

**Authors:** Manidipa Barman, Sanchit Aggarwal, Sarthak Chakrabarti, Sharusa Mandal, Anand S Mishra, Anjali Chaurasia, Diksha Gupta, Prateek Kumar Panda

**Affiliations:** 1 Pediatrics, All India Institute of Medical Sciences, Rishikesh, Rishikesh, IND

**Keywords:** mikito takayasu, adolescent girl, congestive heart failure, dilated cardiomyopathy, takayasu arteritis

## Abstract

We hereby report a 17-year-old adolescent who presented with heart failure with an underlying diagnosis of Takayasu arteritis. Her clinical complaints were intermittent fever, claudication pain in the left upper limb, New York Heart Association (NYHA) grade III dyspnea, and episodes of dizziness for the past two years. On examination, she was unconscious, had absent peripheral pulses, and had severe hypotension. Diagnostic investigations revealed anemia, deranged renal and liver functions, cardiomegaly on chest X-rays, and severe coarctation of the aorta on echocardiography. Further imaging with CT aortography highlighted extensive arterial wall thickening. Laboratory findings included elevated inflammatory markers and negative autoimmune and infectious markers, confirming the diagnosis of large vessel vasculitis (Takayasu arteritis) with heart failure with reduced ejection fraction (LVEF 20-25%) in NYHA class III. The patient was managed with a combination of antihypertensive medications, diuretics, and immunosuppressive therapy. Subsequent follow-up demonstrated improvement in heart failure symptoms and inflammatory markers. This case emphasizes the challenging diagnostic and therapeutic considerations in managing Takayasu arteritis with concurrent cardiovascular complications in the adolescent population.

## Introduction

Takayasu arteritis (TA) is a form of large-vessel vasculitis characterized by persistent granulomatous inflammation in the walls of blood vessels. Alternatively, it is known as 'occlusive thromboarteriopathy,' 'pulseless disease,' or 'Martorell syndrome.' Primarily impacting large vessels such as the ascending aorta, the aortic arch, and its main branches, TA is seen more commonly in young females. The clinical presentation of TA typically includes nonspecific features like hypertension, claudication of limbs, loss of weight, fever, joint pain, and nonspecific arterial pain. Reported cases have documented a variety of clinical manifestations [1]. This case report highlights a rare occurrence of TA in an adolescent girl, where dilated cardiomyopathy with congestive heart failure stands out as the predominant presentation.

## Case presentation

A 17-year-old female adolescent presented with a two-year history of left upper limb pain, four months of dyspnea, two months of generalized body swelling, a ten-day fever, and three days of dizziness. Upon presentation, she was unconscious, with cold extremities, absent peripheral pulses, a heart rate of 90 beats per minute, and an unrecordable blood pressure. She exhibited hypoglycemia (random blood sugar: 38 mg/dl), a respiratory rate of 44 breaths per minute, and an oxygen saturation of 95% on a face mask. Physical examination revealed pallor, facial puffiness, elevated jugular venous pressure, and bilateral pitting edema.

A detailed clinical examination disclosed the absence of left brachial, radial, and bilateral femoral pulses, along with a four-limb blood pressure discrepancy (right arm 167/92 mm Hg, left arm 92/64 mm Hg, right leg 109/84 mm Hg, left leg 86/47 mm Hg). Visible pulsation was noted in the suprasternal area, with a bruit heard over the left carotid, left supraclavicular, and infraclavicular areas. Auscultation revealed audible S1 and S2 with a loud P2 and a pansystolic murmur in the left parasternal area. The liver was palpable 6 cm under the right costal margin, characterized by firmness, smoothness, and non-tenderness.

**Figure 1:**
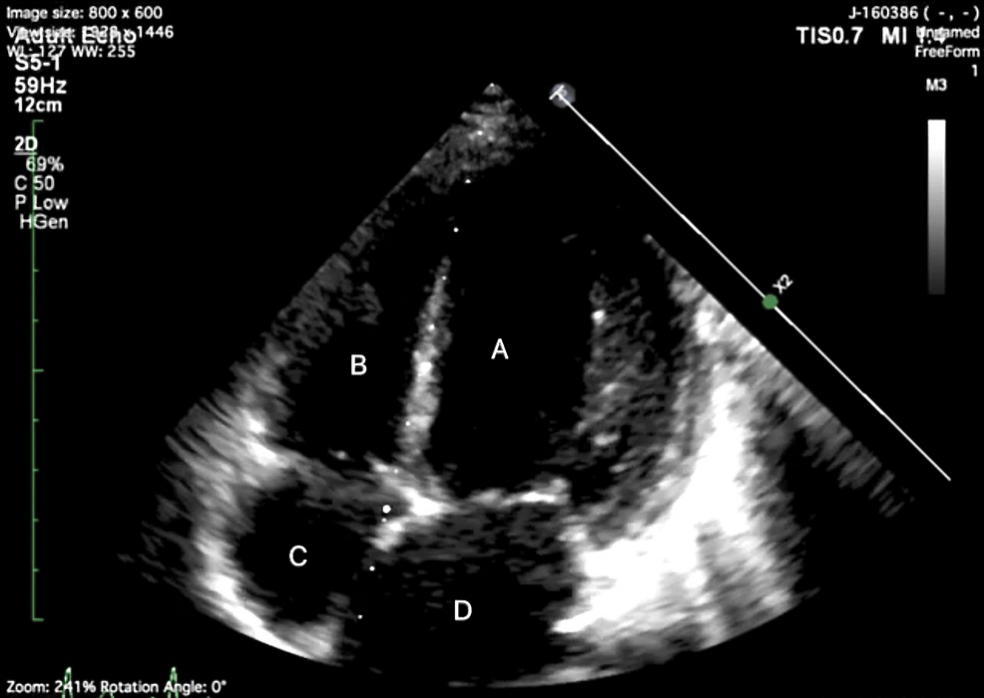
Apical four-chamber view showing left ventricle and left atrium dilatation A: left ventricle; B: right ventricle; C: right atrium and D: left atrium

Investigations unveiled anemia (Hb: 9.9 gm/dl) and abnormal renal function (urea: 41, creatinine: 0.68). Additionally, liver function tests indicated total serum bilirubin (TSB) of 1.27, direct serum bilirubin (DSB) of 0.59, and elevated serum glutamic oxaloacetic transaminase and serum glutamate pyruvate transaminase (SGOT/SGPT) levels of 59/25. Chest X-ray revealed cardiomegaly, while echocardiography depicted severe left ventricular systolic dysfunction (left ventricular ejection fraction: 20-25%), dilated left atrium and left ventricle, moderate mitral regurgitation, moderate tricuspid regurgitation, and severe coarctation of the aorta with a peak gradient of 57 mm Hg (Figure 1). Ultrasound Doppler illustrated diffuse circumferential thickening of the left common carotid artery, subclavian artery, and axillary artery with no color flow and monophasic waves in the left brachial, radial, and ulnar arteries.

CT aortography indicated circumferential wall thickening of the aortic arch and descending thoracic aorta up to the T9 vertebral level. The maximal luminal narrowing was observed at the level of the T5-T6 vertebra, with thickening up to 6 mm and a residual luminal diameter of approximately 3 mm. Wall thickening was also noted along the origin of the celiac trunk, with an attenuated caliber of the celiac trunk and its branches. Laboratory evaluations demonstrated raised inflammatory markers, including a C-reactive protein (CRP) level of 80 mg/L and an erythrocyte sedimentation rate (ESR) of 63 mm/hour. Other laboratory results indicated elevated creatine phosphokinase-MB (CPK-MB) (108 U/L) and ferritin (130), while tests for antinuclear antibody (ANA), anti-neutrophilic cytoplasmic antibody (ANCA), HbsAg, anti-HCV antibody, anti-HIV antibody, and Mantoux were negative. The diagnosis was established as large vessel vasculitis: Takayasu aortoarteritis with heart failure with reduced ejection fraction (HFrEF; LVEF 20-25%) in NYHA class III.

**Figure 2:**
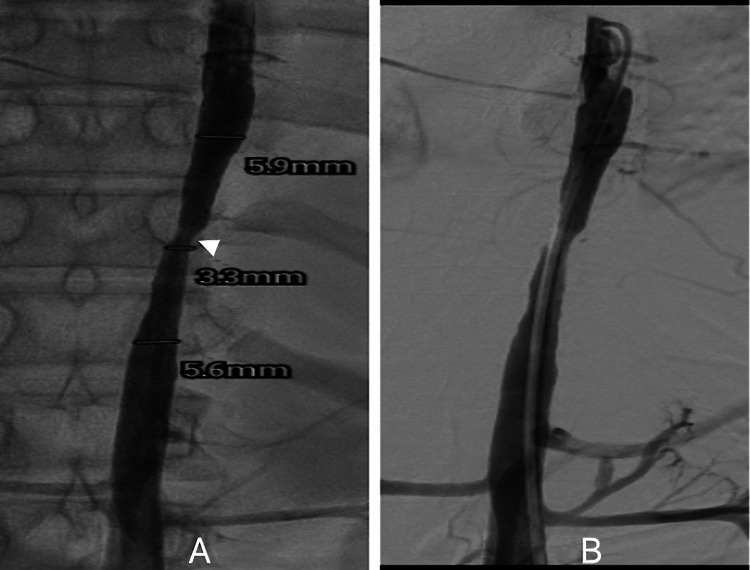
Percutaneous transfemoral balloon angioplasty A: narrowing of the thoracic aorta (solid arrowhead); B: balloon angioplasty being performed

The patient received ramipril at a dose of 7.5 mg/day, metoprolol at a dose of 1 mg/kg/day, and nicardipine for hypertension. Oral diuretics (torsemide and spironolactone) were administered to address congestive heart failure symptoms, while a combination of oral prednisolone (1 mg/kg/day) with mycophenolate mofetil was given for vasculitis. After one month of medical intervention, the patient exhibited only a slight reduction in heart failure with persistent stenotic symptoms; hence, percutaneous trans-femoral balloon angioplasty was performed for stenotic complications (Figure 2).

Follow-up evaluations of inflammatory markers showed CRP and ESR levels of 0.44 and 22, respectively. The child is currently under follow-up and doing well.

## Discussion

Takayasu arteritis is an uncommon, chronic, and progressive inflammatory ailment affecting large and medium-sized arteries. The condition results in gradual fibrosis and constriction of the arterial lumen, leading to the destruction of the arterial media and the formation of aneurysms. It predominantly impacts young females in their second and third decades, with a female-to-male ratio of 6.4:1 in India and 9:1 in Japan [2]. First documented by Japanese ophthalmologist Mikito Takayasu in 1908, the disease initially manifested with retinal changes in a young female.

As per the American College of Rheumatology, the six diagnostic criteria for Takayasu arteritis include the onset of the disease before the age of 40, extreme claudication, a diminished pulse in the brachial artery, bruit in the subclavian arteries or aorta, a blood pressure difference exceeding 10 mm Hg, and abnormal angiography.

A diagnosis of Takayasu arteritis is established if at least three of the above six criteria are met [1]. In our case, the patient exhibits all six criteria, confirming the diagnosis of Takayasu arteritis.

The cause of Takayasu arteritis is understood to involve an autoimmune mechanism, with a connection noted with specific human leukocyte antigen (HLA) types such as A2, A9, B35, B52, and DR4. An increase in the levels of pro-inflammatory cytokines, including interleukin (IL)-6, IL-8, IL-9, IL-12, IL-17A, IL-18, interferon (IFN) gamma, and tumor necrosis factor (TNF) alpha, plays a crucial role in the development of the disease [3,4]. The clinical signs of Takayasu arteritis range from having no apparent symptoms to nonspecific or severe manifestations.

There are three stages of Takayasu arteritis. The first stage is the early systemic/pre-pulseless stage. This stage is characterized by nonspecific systemic symptoms like mild fever, night sweats, fatigue, malaise, joint pains, myalgia, and weight loss. Diagnosis is challenging during this phase. The second is the vascular-inflammatory stage. In this stage, there are symptoms related to vascular inflammation and insufficiency, such as limb claudication, hypertension, and arterial bruits, as well as manifestations in the ocular, dermatologic, and neurological areas. The third is the burnout stage. This stage involves the fibrosis of the affected vessels [5].

Takayasu arteritis is documented as the etiology for renovascular hypertension in 28%-75% of cases in India, congestive heart failure in 76% of cases, aortic regurgitation in 20-24% of cases, and dilated cardiomyopathy in only 5% of cases [6]. Our case is an adolescent female who presented with congestive heart failure with dilated cardiomyopathy and absent arterial pulses in the left upper and bilateral lower limbs. According to the American College of Rheumatology diagnostic criteria, our case fulfilled all six criteria and was hence diagnosed as Takayasu arteritis.

## Conclusions

This case demonstrates a rare case of Takayasu arteritis in an adolescent female presenting with congestive heart failure and dilated cardiomyopathy. With this case report, we want to emphasize that any adolescent or young patient with heart failure should be screened for systemic vasculitis to reduce complications and improve overall quality of life in cases of large vessel vasculitis.
